# The ubiquitin-proteasome system in neurodegenerative diseases: precipitating factor, yet part of the solution

**DOI:** 10.3389/fnmol.2014.00070

**Published:** 2014-07-31

**Authors:** Nico P. Dantuma, Laura C. Bott

**Affiliations:** ^1^Department of Cell and Molecular Biology, Karolinska InstitutetStockholm, Sweden; ^2^Neurogenetics Branch, National Institute of Neurological Disorders and Stroke, National Institutes of HealthBethesda, MD, USA

**Keywords:** neurodegeneration, ubiquitin, proteasome, proteolysis, protein quality control

## Abstract

The ubiquitin-proteasome system (UPS) has been implicated in neurodegenerative diseases based on the presence of deposits consisting of ubiquitylated proteins in affected neurons. It has been postulated that aggregation-prone proteins associated with these disorders, such as α-synuclein, β-amyloid peptide, and polyglutamine proteins, compromise UPS function, and delay the degradation of other proteasome substrates. Many of these substrates play important regulatory roles in signaling, cell cycle progression, or apoptosis, and their inadvertent stabilization due to an overloaded and improperly functioning UPS may thus be responsible for cellular demise in neurodegeneration. Over the past decade, numerous studies have addressed the UPS dysfunction hypothesis using various model systems and techniques that differ in their readout and sensitivity. While an inhibitory effect of some disease proteins on the UPS has been demonstrated, increasing evidence attests that the UPS remains operative in many disease models, which opens new possibilities for treatment. In this review, we will discuss the paradigm shift that repositioned the UPS from being a prime suspect in the pathophysiology of neurodegeneration to an attractive therapeutic target that can be harnessed to accelerate the clearance of disease-linked proteins.

## Introduction

Proteinopathies form a large group of pathologies that are characterized by the presence of abnormally folded proteins in affected cells (Carrell and Lomas, 1997). Among those diseases are hereditary neurodegenerative disorders, such as Huntington's disease (HD), spinal and bulbar muscular atrophy (SBMA), and several forms of autosomal dominant spinocerebellar ataxias (SCAs; types 1-3, 6, 7, 17), caused by polyglutamine (polyQ) repeat expansions in unrelated proteins (Orr and Zoghbi, 2007; La Spada and Taylor, 2010). The observation that the expanded polyQ repeat renders proteins prone to aggregation has raised the idea that members of this disease family may share a common pathogenic mechanism (Scherzinger et al., 1997). It has further been suggested that related pathogenic events may be an underlying cause in other neurodegenerative diseases characterized by the presence of protein aggregates, such as Alzheimer's disease (AD), Parkinson's disease (PD) and amyotrophic lateral sclerosis (ALS) (Sherman and Goldberg, 2001). The technical advantage of working with monogenic polyQ diseases has brought together scientists from a broad variety of research disciplines who have studied the effects of the pathogenic proteins on a wide spectrum of cellular processes in cultured cells and animal models.

Since the discovery of repeat expansions as a genetic basis of hereditary neurodegenerative diseases more than two decades ago (La Spada et al., 1991), we have learned that polyQ proteins have an impact on diverse cellular processes such as transcription, transport, neuronal function, and viability. The long list of cellular functions affected by these proteins suggests that the disease-linked proteins disturb one or more systems central to these processes, causing many downstream pathways to collapse during the course of pathology. The ubiquitin-proteasome system (UPS) has received particular attention in the study of neurodegenerative disorders due to its role as a critical regulator of protein homeostasis in eukaryotic cells. It keeps the cellular environment free of misfolded, defective, and aggregation-prone proteins, which have been found to accumulate in neurodegenerative diseases (Ciechanover and Brundin, 2003). The protein quality control function of the UPS is, however, only one of the essential processes that engages this multitasking proteolytic system which governs also cell cycle progression and induction of apoptosis (Hershko and Ciechanover, 1998). It has been proposed that the vast amounts of aggregation-prone polyQ proteins may overwhelm the UPS and compromise other essential functions of the machinery required for maintaining cellular homeostasis (Mayer et al., 1989). According to this model, blockade of the UPS by the disease protein would result in global accumulation of proteasome substrates and thus provides an explanation for the broad and diverse effects on cellular homeostasis in affected cells.

Many laboratories have addressed the UPS dysfunction hypothesis over the last decade and it has become evident that UPS activity is preserved in the majority of neurodegenerative disorders. In the course of these studies, adaptive cellular responses have been identified that help to alleviate the burden of aggregation-prone proteins to keep ubiquitin-dependent proteolysis operative. Here we will discuss the transition of our view on the UPS from a dysfunctional system that catalyzes cytotoxicity in neurodegenerative diseases to a powerful proteolytic system that may be exploitable in therapeutic strategies aimed at clearing aggregation-prone proteins from cells.

## The ubiquitin-proteasome system as prime suspect

The UPS, which is the principal pathway for the clearance of short-lived, damaged, and misfolded proteins in the nucleus and cytoplasm, consists of two separate, consecutive steps: ubiquitylation and proteasomal degradation (Hershko and Ciechanover, 1998; Kleiger and Mayor, 2014). An enzymatic cascade composed of ubiquitin activator, conjugase, and ligase catalyzes the covalent attachment of ubiquitin to a substrate protein. Ubiquitin is conjugated via its carboxy-terminal glycine to an internal lysine (Lys) residue or, less commonly, to the free amino (N) terminus of the substrate (Pickart, 2001). Multiple rounds of ubiquitylation lead to the formation of a polyubiquitin chain, which can function as a signal for degradation by the proteasome, a multi-protein complex consisting of a 20S core particle and 19S regulatory particles, at one or both ends. The active sites responsible for the chymotrypsin-like, trypsin-like, and caspase-like activities of the proteasome are situated in the interior surface of the 20S core particle, thereby shielding their proteolytic activities from the rest of the cellular proteome (Bedford et al., 2010). The proteasome unfolds substrates and threads the polypeptide chains through the inner channel, where they are cleaved into short peptides (Bhattacharyya et al., 2014). Following their release from the barrel, peptides are rapidly processed into amino acids by cellular aminopeptidases and recycled (Reits et al., 2003).

**Figure 1:**
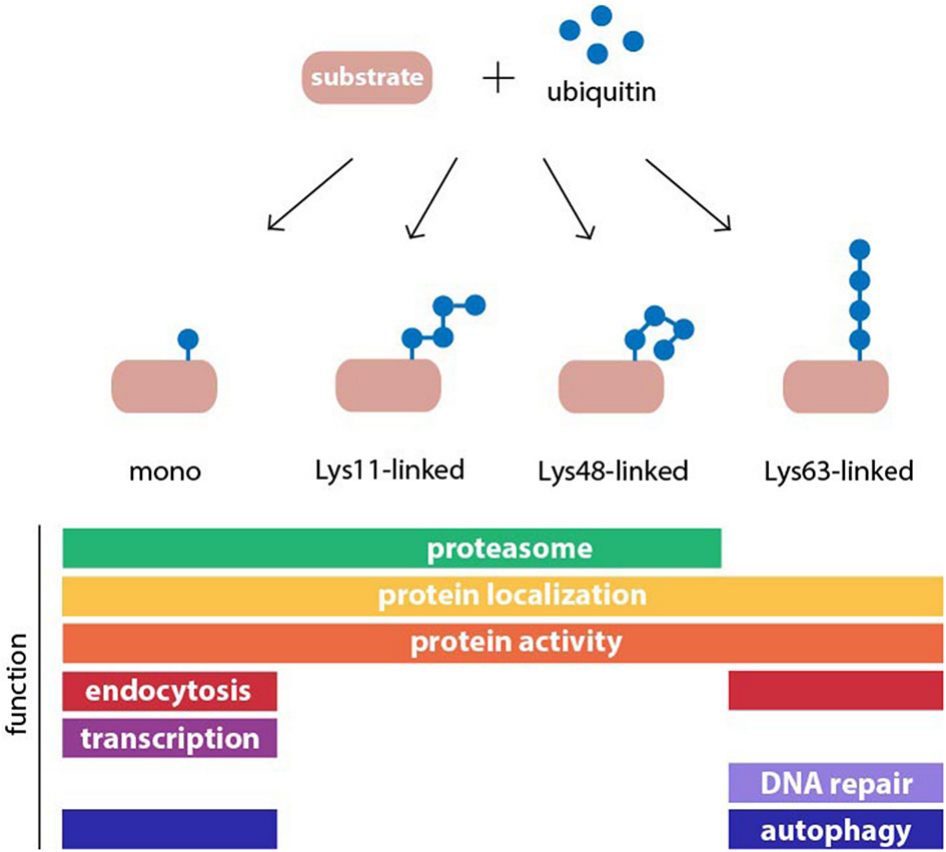
**Structure and function of common ubiquitin modifications**. Ubiquitin may be conjugated to protein substrates as either a monomer or a polymeric chain, in which one of seven internal lysine (Lys) residues of ubiquitin, or the N-terminal methionine, serves as an acceptor for additional ubiquitin moieties. The type of polyubiquitin linkage dictates the topology of the resulting chain. Ubiquitin modifications can regulate protein function or act as a signal in many cellular processes. Examples for functions of monoubiquitylation, and homogenous Lys11-, Lys48-, and Lys63-linked polyubiquitin chains are shown.

Ubiquitylation has many other roles in cells besides proteasomal degradation (Figure 1). The destiny of a given substrate protein is determined by the type of ubiquitin assembly to which it is connected. This is possible because ubiquitin contains seven Lys residues in its amino acid sequence at positions 6, 11, 27, 29, 33, 48, and 63, which can serve as acceptors for additional ubiquitin monomers in the construction of polyubiquitin chains (Komander and Rape, 2012). As a result, many different chain topologies can be formed, which are recognized by specific ubiquitin-binding adaptors in the relevant cellular pathways. Chain topology and substrate specificity are determined by a large spectrum of ubiquitin-ligating and -modifying enzymes, whose expression levels and activities are tightly regulated in a tissue-, cell-, and compartment-specific manner. Several different types of polyubiquitin linkages target substrates to the proteasome, such as Lys11 and Lys29 in addition to the canonical Lys48-linked chains, as well as conjugation of a single ubiquitin molecule (Kravtsova-Ivantsiv and Ciechanover, 2012). Mono- and polyubiquitin chains also regulate non-proteolytic functions in cells, such as protein activity and localization (Seet et al., 2006). While most ubiquitin chains can target proteins for proteasomal degradation, it has been shown that Lys63-linked ubiquitin chains are the only modification that do not behave as a proteasome targeting signal *in vivo* (Nathan et al., 2013). Instead these chains play pivotal roles in signaling, endocytosis, and DNA repair. More recently, this chain topology has been implicated in macroautophagy (Kraft et al., 2010), a pathway that targets cytoplasmic proteins and organelles for degradation in lysosomes (Nakatogawa et al., 2009). Therefore, the UPS is highly interconnected with other proteolytic and non-proteolytic cellular processes at multiple levels, whereby it controls many diverse functions in cells.

Given the vast amount of proteins that are involved in the UPS, it is perhaps not surprising that some of them have been genetically linked to neurodegenerative disorders (Ciechanover and Brundin, 2003). On first sight, the finding that mutations in genes encoding components of the UPS can cause or predispose for neurodegeneration supports the notion of inefficient ubiquitin-dependent proteolysis as a shared pathogenic mechanism. However, it should not be overlooked that the UPS and, in particular, the ubiquitin-targeting step are connected to many different processes besides proteasomal degradation. Several cases of neurodegeneration-linked mutations in UPS components are now known to affect ubiquitin-dependent processes that do not target proteins to the proteasome, while the UPS remains largely operative.

One of the best-studied examples of a UPS component that has been linked to neurodegeneration is the ubiquitin ligase Parkin, mutations in which cause an autosomal recessive juvenile-onset PD (Kitada et al., 1998). Early studies revealed that Parkin can target endoplasmic reticulum (ER)-derived proteins (Yang et al., 2003) and polyQ proteins for proteasomal degradation (Tsai et al., 2003) suggesting that a defect in the UPS-mediated protein quality control may be responsible for this pathology. However, during recent years it has become apparent that Parkin is also involved in the autophagy pathway that results in the degradation of dysfunctional mitochondria in lysosomes, a process known as mitophagy (Ashrafi and Schwarz, 2013). Parkin cooperates with the mitochondrial kinase PINK1, which has also been linked to PD, thus strengthening the genetic association between mitophagy and neurodegeneration (Clark et al., 2006; Park et al., 2006). Another theory suggests that Lys63-linked polyubiquitin chains generated by Parkin may be important for targeting aggregation-prone proteins to inclusions bodies (IBs) (Olzmann et al., 2007). Although the possibility remains that UPS dysfunction contributes to PD, recent studies point to autophagy-related processes as central to the pathology caused by Parkin mutations.

Another example is ubiquilin, a ubiquitin-binding shuttle factor that is involved in escorting polyubiquitylated proteins to the proteasome for degradation (Elsasser and Finley, 2005). Overexpression of ubiquilin has a neuroprotective effect in mice expressing a fragment of the polyQ protein causative for HD, huntingtin (El Ayadi et al., 2012). A single nucleotide polymorphism (SNP) that causes alternative splicing of the ubiquilin transcript predisposes for late-onset AD (Bertram et al., 2005). Given the function of ubiquilin as a shuttling substrate receptor in the UPS, it is tempting to speculate that the SNP in ubiquilin alters the ability of cells to recognize and destroy misfolded proteins. However, the AD-linked ubiquilin variants do not cause a general block of the UPS, but instead, were found to selectively cause accumulation of presenilin-1, which is involved in amyloid precursor protein (APP) processing (Viswanathan et al., 2011). Moreover, ubiquilin-1 functions as a molecular chaperone that regulates trafficking and processing of APP, which has been linked to its ability to stimulate polyubiquitylation of APP with non-proteolytic Lys63-linked polyubiquitin chains (El Ayadi et al., 2012). Together this suggests that while ubiquilin-1 may regulate the production of β-amyloid peptide at multiple levels in a ubiquitin-dependent fashion, the variants linked to AD do not seem to trigger a general failure of the UPS.

The ubiquitin-selective chaperone valosin-containing protein (VCP) has also attracted attention from researchers who study the link between the UPS and neurodegenerative disorders. Mutations in VCP cause multisystem proteinopathy (MSP), with among its spectrum of symptoms frontotemporal dementia (Watts et al., 2004), as well as the motor neuron disease amyotrophic lateral sclerosis (ALS) (Johnson et al., 2010). VCP is critical for proteasomal degradation of certain proteins and it is believed that it does so by means of its ATP-dependent chaperone activity that can unfold or segregate proteasome substrates from their environment (Stolz et al., 2011). However, ubiquitin-dependent functions of VCP are not limited to proteasomal degradation (Dantuma and Hoppe, 2012; Meyer et al., 2012) and include ubiquitin-selective autophagy (Ju et al., 2009; Tresse et al., 2010), the clearance of stress granules (Buchan et al., 2013), mitochondrial integrity (Bartolome et al., 2013), Parkin-dependent mitophagy (Kim et al., 2013), and the DNA damage response (Acs et al., 2011; Meerang et al., 2011). Overexpression of VCP mutants linked to MSP and ALS did not inhibit the UPS (Tresse et al., 2010), whereas some of the VCP-mediated events mentioned above were altered (Ju et al., 2009; Tresse et al., 2010; Bartolome et al., 2013; Fujita et al., 2013; Kim et al., 2013), suggesting that the pathology caused by mutant VCP likely involves essential ubiquitin-dependent processes that are different from proteasomal degradation.

A unique example of a protein responsible for a polyQ neurodegenerative disease that is involved in ubiquitin-dependent proteasomal degradation is ataxin-3. It functions as a deubiquitylation enzyme that reverses the ubiquitin mark on proteins by disassembling Lys48- and Lys63-linked polyubiquitin chains with a preference for the latter (Winborn et al., 2008). Expansion of the polyQ repeat that resides in ataxin-3 are the underlying cause for the most common form of autosomal dominant SCA, known as SCA-3 or Machado-Joseph disease. Notably, ataxin-3 physically interacts with VCP and regulates proteasomal degradation of ER-derived substrates (Wang et al., 2006; Zhong and Pittman, 2006). Overexpression of either wild-type or polyQ-expanded ataxin-3 compromises the functionality of the UPS resulting in increased levels of proteasome substrates (Burnett et al., 2003). Interestingly, non-expanded ataxin-3 is also a known suppressor of polyQ toxicity in models of SCA-3 and other polyQ diseases (Warrick et al., 2005). Although the reason for this protective effect is poorly understood, it may be attributed to the ability of ataxin-3 to stimulate sequestration of misfolded proteins (Burnett and Pittman, 2005). Finally, ataxin-3 regulates the ubiquitylation status of Parkin and stimulates its degradation by autophagy, linking also this protein to other ubiquitin-dependent systems (Durcan et al., 2011). This study, like the previous examples, suggests a complex relationship between the UPS and neurodegeneration and shows that the effects of disease-associated proteins on ubiquitin-dependent proteolysis are difficult to predict.

Two important lessons can be drawn from the afore-mentioned examples. First, each one of these examples underscores the prevalent notion that the UPS is tightly connected to neurodegenerative disorders. The fact that unrelated diseases share the presence of causative mutations in UPS components leaves little doubt that the UPS plays a central role in these pathologies. Second, they illustrate that care should be taken when extrapolating genetic data to disease mechanisms. Thus, the general view that a compromised UPS is responsible for the typical accumulation of misfolded proteins in neurodegenerative diseases is problematic due the complex and multilayered connection between the UPS and a variety of cellular functions. The fact that a large number of essential cellular functions other than protein quality control require ubiquitin conjugation or proteasomal degradation leaves the possibility open that the role of the UPS in these pathologies may be largely unrelated to ubiquitin-dependent protein quality control. A definite answer on whether, and if so, to what extent, impairment of the UPS contributes to the development of neurodegenerative diseases can only be obtained by empirically assessing the functional status of the UPS in each of these diseases.

## Ubiquitin-positive inclusions

A hallmark of neurodegenerative diseases is the accumulation of abnormal proteins in insoluble deposists, or IBs. The idea that impaired clearance of misfolded proteins may be central to neurodegenerative diseases originates in part from the observation that IBs contain ubiquitin, proteasome subunits, and other UPS components (Cummings et al., 1998). Mori et al. who first described the presence of ubiquitin in IBs, already speculated that either the protease responsible for degrading ubiquitylated proteins (which was not known at that time to be the proteasome) was dysfunctional, or that ubiquitylated proteins residing in the inclusions resisted degradation (Mori et al., 1987).

For a long time, the nature of IBs has been under debate (Sisodia, 1998). While some researchers argued that they were innocent bystanders, others favored the view that IBs were directly implicated in the cellular pathology caused by aggregation-prone proteins (Sherman and Goldberg, 2001). One hypothesis that has appeared in different forms with various key players suggests that physical entrapment of proteins in IBs removes functional proteins from critical cellular processes. With respect to the UPS and chaperones, one can picture how sequestration may compromise their household function and elicit cellular pathology. According to yet another model, IBs are part of the cellular defense mechanism that protects cells against toxicity by functioning as a sink for misfolded proteins. A wealth of data in support for the latter option comes from cell biologists who found that ectopic overexpression of abnormal proteins triggers a sequence of reactions that results in the deposition of the misfolded proteins into specific perinuclear structures, which were coined aggresomes (Wojcik et al., 1996; Johnston et al., 1998; Garcia-Mata et al., 1999). These findings argue against the view that IBs are formed by passive aggregation, but instead require active, ATP-dependent processes in cells (Kopito, 2000). Moreover, related protein deposits have been documented in budding yeast, which suggests that this cellular response is conserved throughout evolution and serves cytoprotective functions in both unicellular and multicellular eukaryotic organisms (Kaganovich et al., 2008). In dividing cells, IBs are distributed asymmetrically to daughter cells during mitosis ensuring the generation of healthy progeny devoid of proteineous deposits (Rujano et al., 2006).

The most compelling evidence supporting a role of IBs as an adaptive response comes from live cell recordings, which show that IB formation correlates with increased survival in primary neurons expressing a mutant huntingtin fragment (Arrasate et al., 2004). This is consistent with the finding that reducing the load of IBs, achieved by genetically inhibiting ubiquitylation, enhances polyQ-mediated neurodegeneration in a SCA-1 mouse model (Cummings et al., 1999). Inclusions are reversible and highly dynamic structures, as switching off expression of a mutant huntingtin fragment (Yamamoto et al., 2000) or mutant ataxin-1 (Zu et al., 2004) in conditional mouse models results in the clearance of ubiquitin-positive IBs. When it comes to the UPS components and chaperones associated with IBs, it has been demonstrated that these factors do not appear to be physically trapped in IBs (Kim et al., 2002; Stenoien et al., 2002; Holmberg et al., 2004). A recent study even suggests that proteasomes play an active role in maintaining or dissolving these structures (Schipper-Krom et al., 2014). Furthermore, POH1/Rpn11, a proteasome subunit with deubiquitylation activity, stimulates the formation of IBs by generating free ubiquitin chains in proximity of the aggregated proteins and results in the recruitment of HDAC6 (Hao et al., 2013), which orchestrates ubiquitin-dependent transport of proteins to the aggresome (Kawaguchi et al., 2003). Altogether, these findings support a protective function of IBs in cells facing large amounts of misfolded proteins. Increasing evidence suggests that IBs may act as a hub for misfolded and aggregated proteins and redirect them to alternative destruction mechanisms, which will be discussed later.

## Proteasome activity in neurodegenerative diseases

One possibility to get a better insight into the functionality of the UPS is by assessing the individual enzymatic activities involved in ubiquitin-dependent proteasomal degradation (Lindsten and Dantuma, 2003). Ubiquitylation, on one hand, is the net result of a large family of enzymes that are involved in proteolytic and non-proteolytic processes and are therefore not straight-forward to address or to interpret. The proteasome, on the other hand, is the final destination of all ubiquitylated substrates to be degraded and creates a bottleneck in the UPS pathway. Its function is readily traceable to the individual proteolytic subunits whose activities can be measured by using specific fluorogenic substrates (Kisselev and Goldberg, 2005) or activity probes (Verdoes et al., 2006). It is therefore not surprising that the proteasome has received a lot of attention in studies that assess the functionality of the UPS in neurodegenerative diseases. However, correlation of proteasome activity measurements and UPS impairment in neurodegenerative diseases is complicated by the fact that it is presently unknown to what extent altered proteasome activity affects the overall flux of degradation of ubiquitylated substrates. Fibroblasts derived from mice with a heterozygous deletion of PSMC1/Rpt2, one of the ATPase subunits of the 19S regulatory particle, develop without obvious defects despite reduced proteasome function (Rezvani et al., 2012). Moreover, proteasome activity can be regulated through expression of individual subunits. For example, increasing the amount of active proteasomes, either through overexpression of the 20S core component PSMB5/β 5 responsible for the chymotrypsin-like activity or the proteasome assembly chaperone hUMP1/POMP, improves resistance to oxidative stress insults in human fibroblasts (Chondrogianni et al., 2005; Chondrogianni and Gonos, 2007). Likewise, levels of the PSMD11/Rpn6 proteasome subunit, which stabilizes the interaction between the regulatory and core particle and facilitates ubiquitin-dependent degradation (Pathare et al., 2012), can regulate proteasome activity (Vilchez et al., 2012a,b).

Translating how certain levels of proteasome inhibition will jeopardize the functional status of ubiquitin-dependent proteasomal degradation poses another unresolved problem in assessing the status of the UPS through proteasome activity measurements. Experiments with yeast strains expressing mutant proteasome subunits suggest that the proteolytic sites are non-redundant and vary in their contribution to overall protein degradation (Rubin et al., 1998). The chymotrypsin-like activity of the proteasome needs to be reduced by more than 80 percent in human cells before the clearance of ubiquitylated proteins becomes noticeably delayed (Dantuma et al., 2000; Bence et al., 2001). Even when this threshold is not reached, a lower degree of inhibition of the chymotrypsin-like activity does, however, impair the cells' ability to deal with an acute increase in the flux of ubiquitylated proteins during stress conditions (Dantuma et al., 2000). In mammalian cells, individual proteasome activities also differ in their contribution to overall protein degradation since the caspase-like activity can be chemically ablated without affecting the ability of cells to clear ubiquitylated proteins (Myung et al., 2001). Therefore, the differential contribution of individual proteolytic activities, the high level of redundant proteasome activity, and indirect effects of the stress status, which determine the load of ubiquitylated substrates, complicate the interpretation of proteasome activity measurements when it comes to the functional status of the UPS.

Initial studies have measured proteasome activity in postmortem human tissues and reported decreased activity in neurodegenerative disorders (Table 1). However, follow-up experiments with purified proteasomes, cell lines, and animal models have not always been consistent with those early reports. While in some studies a decrease in proteasome activity has been reported, others have found that the activity is unchanged or even increased (for references see Table 1). Increased proteasome activity could be an adaptive response to the augmented load of misfolded proteins in these diseases but there are also alternative explanations. In this respect, it is important to point out there is a set of alternative proteolytic subunits that can replace the constitutive proteases in the proteasome in response to interferon γ (Kniepert and Groettrup, 2014). The resulting immunoproteasome plays an important role in generating peptide fragments for antigen display by major histocompatibility complex class I proteins on the cell surface. Thus, changes in the activity of the proteasome may also be attributed to induction of immunoproteasomes in inflammatory response, which are commonly observed in neurodegenerative diseases (Czirr and Wyss-Coray, 2012). Indeed induction of immunoproteasome subunits have been reported in mouse models of polyQ disorders (Diaz-Hernandez et al., 2003) and other neurodegenerative diseases (Mishto et al., 2006; Cheroni et al., 2009; Orre et al., 2013).

**Table 1:** **Overview of measurements of the proteolytic activity of the proteasome in neurodegenerative disease models**.

**Disease**	**Protein context**	**Model system**	**Reduced activity?**	**References**
Polyglutamine disease	Polyglutamine GFP fusion	COS-1 cell line	No	Michalik and Van Broeckhoven, 2004
		SHSY5Y cell line	No	Ding et al., 2002
	Htt fragment	Purified proteasomes	Yes/No^a^	Diaz-Hernandez et al., 2006
			No	Bennett et al., 2005; Hipp et al., 2012
		Neuro2a cell line	Yes	Jana et al., 2001
		ST14A cell line	Yes	Seo et al., 2007
		R6/2 mice	No	Bett et al., 2006; Wang et al., 2008; Maynard et al., 2009
		HD94 mice	No	Diaz-Hernandez et al., 2003
	Ataxin-7	Sca7^266Q/5Q^ mice	No	Bowman et al., 2005
	Androgen receptor	AR97Q mice	No	Tokui et al., 2009
	HD postmortem brain; patient fibroblasts		Yes	Seo et al., 2004
Polyalanine disease	PABPN1	OPMD A17.1 mice	No	Trollet et al., 2010
Alzheimer's disease	β-amyloid peptide	Purified proteasomes	Yes	Gregori et al., 1995; Tseng et al., 2008
			No	Kristiansen et al., 2007
		B103 cell line	Yes	Song et al., 2003
		GT1 and N2aPK-1 cell lines; cerebellar granule neurons	No	Kristiansen et al., 2007
		Primary astrocytes and neurons	Yes	Lopez Salon et al., 2003
		Neuro2a and N9 cell lines; primary astrocytes and microglia	No	Orre et al., 2013
	Tg2576 mice		Yes	Oh et al., 2005; Almeida et al., 2006
	3xTg-AD mice		Yes/No^b^	Tseng et al., 2008
	APP/PS1 mice		Yes/No^c^	Aso et al., 2012
			No	Orre et al., 2013
	AD postmortem brain		Yes	Keller et al., 2000; Lopez Salon et al., 2000; Keck et al., 2003; Mishto et al., 2006
			No	Orre et al., 2013
Parkinson's disease	Wild type α-synuclein	Purified proteasomes	Yes	Snyder et al., 2003
		HEK293 and BE-M17 cell lines	Yes	Snyder et al., 2003
		hwα-SYN-5 mice	Yes	Chen et al., 2006
	Mutant α-synuclein	PC12 cell line	Yes	Stefanis et al., 2001; Tanaka et al., 2001
		hm^2^α-SYN-39 mice	Yes	Chen et al., 2006
	Parkin	Parkin loss-of-function flies; Parkin null-mice	Yes	Um et al., 2010
	LRRK2	HeLa cell line	No	Lichtenberg et al., 2011
	PD postmortem brain		Yes	McNaught and Jenner, 2001; McNaught et al., 2003
ALS	Mutant SOD1	Purified proteasomes	No	Kristiansen et al., 2007
		Neuro2A cell line	Yes	Urushitani et al., 2002
		NT-2, SK-N-MC, and SH-SY5Y cell lines	No	Lee et al., 2001; Casciati et al., 2002; Aquilano et al., 2003
		GT1 and N2aPK-1 cell lines; cerebellar granule neurons	No	Kristiansen et al., 2007
ALS/IBMPFD	VCP	Purified proteasomes	Yes	Gitcho et al., 2009
Retinal degeneration	Transducin γ-subunit	G^-/-^_γ1_mice	No	Lobanova et al., 2013
Prion disease	Prion protein	Purified proteasomes	Yes	Kristiansen et al., 2007; Deriziotis et al., 2011
		GT1 and N2aPK-1 cell lines; cerebellar granule neurons	Yes	Kristiansen et al., 2007

a *Filamentous htt but not inclusions isolated from HD94 mice was shown to inhibit the 26S proteasome in vitro without affecting 20S proteasome function*.

b *Decreased proteasome activity observed only at early time points; no difference compared with wild type at late disease stages*.

c *Decreased chymotryptic activity, but no decreased tryptic- and caspase-like activity*.

An exceptional case, in which the presence of an aggregation-protein has been mechanistically linked to inhibition of the proteasome, is the prion protein, which causes when misfolded the fatal, transmissible neurodegenerative disorder Creutzfeld-Jacob disease. Wild-type prion proteins residing in the cytosolic compartment of cells are efficiently degraded in a ubiquitin-dependent fashion (Yedidia et al., 2001) whereas cytosolic mutant prion proteins form aggresomes, which is accompanied by signs of apoptotic cell death (Kristiansen et al., 2005). It has been shown that oligomers of mutant prion protein effectively inhibit the activity of the proteasome *in vitro* and *in vivo* (Kristiansen et al., 2007) through direct binding of oligomers to the 20S proteasome core particle, which results in stabilization of a closed confirmation of the proteasome (Deriziotis et al., 2011). As a consequence, prion protein inhibits proteolysis by preventing substrate access to the proteasome core.

The main constituent of the Lewy bodies in PD, α-synuclein, has also been shown to bind to proteasomes (Snyder et al., 2003) and inhibit UPS function *in vitro* and *in vivo* (Stefanis et al., 2001; Snyder et al., 2003; Chen et al., 2006). Cytoplasmic amyloid β peptide can also interact with the proteasome (Gregori et al., 1995), however, its effect on proteasome activity is debated (for references see Table 1). Moreover, it is not clear if proteasome inhibition by α-synuclein or β-amyloid is mechanistically related to that of prion proteins.

For polyQ proteins, it has been shown that ubiquitylated filamentous aggregates of mutant huntingtin isolated from mouse or human brain samples can selectively inhibit the proteolytic activity of the proteasome (Diaz-Hernandez et al., 2006). We will discuss below that several studies suggest that this inhibitory activity of huntingtin aggregates does not compromise the UPS *in vivo* (Bett et al., 2009; Maynard et al., 2009; Ortega et al., 2010). Moreover, mutant huntingtin fragments have not been found to inhibit degradation of ubiquitylated substrates by the 26S proteasome *in vitro* (Hipp et al., 2012). Whether or not polyQ proteins can be degraded by the mammalian proteasome has been the subject of a considerable debate (Venkatraman et al., 2004; Pratt and Rechsteiner, 2008; Juenemann et al., 2013). Puromycin-sensitive aminopeptidase has been identified as the main cytosolic protease to efficiently clear expanded polyQ peptides generated by the proteasome *in vitro* (Bhutani et al., 2007). It has been hypothesized that proteasome-derived polyQ fragments may enhance aggregate formation (Venkatraman et al., 2004; Raspe et al., 2009), but it is unclear whether these species are actually generated *in vivo*. In cultured cells, aggregation of mutant huntingtin fragment is exacerbated following treatment with the proteasome inhibitor lactacystin (Waelter et al., 2001). Moreover, enhancing UPS-mediated clearance of polyQ proteins reduces their levels and toxicity, suggesting that the net outcome of accelerated proteasomal degradation is beneficial in polyQ diseases (Verhoef et al., 2002; Michalik and Van Broeckhoven, 2004).

## Functional status of the UPS in neurodegenerative diseases

UPS functionality, which describes the relative rate at which cells ubiquitylate and degrade proteins at a given time, can be addressed in cells and tissues in several ways. These approaches are based on the assumption that cells that cannot maintain a constant flux through the UPS will gradually build up ubiquitylated proteasome substrates irrespective of their nature. As explained earlier, the relationship between UPS activity and levels of substrates is often complex. Reduced proteasome activity does not necessarily lead to functional impairment of the UPS as long as the activity is sufficient to process ubiquitylated proteins targeted for destruction. *Vice versa*, ubiquitin-dependent proteolysis in cells can be severely impaired despite the presence of a normal proteasome activity profile. Furthermore, cells may mobilize compensatory mechanisms to assist a suboptimal but functioning UPS.

A straight-forward approach to assess UPS activity in cells or tissues measures the abundance or quality of ubiquitylated proteins. However, ubiquitylation is involved in many cellular processes other than proteasomal degradation (Komander and Rape, 2012) and it is therefore difficult to extrapolate whether a possible build up in ubiquitylated proteins is a consequence of UPS impairment or reflects other changes in the ubiquitin homeostasis (Groothuis et al., 2006). Substrate flux through the UPS can be assessed by determining the levels or half-lives of cellular proteins. An important caveat with this approach is that the degradation of endogenous proteasome substrates is often tightly regulated and likely to change depending on internal or external cues. Particularly in the context of neurodegenerative diseases, altered degradation rates of endogenous substrates likely reflect functional differences in protein stabilization rather than changes in overall UPS activity. This problem can be circumvented by the use of reporter substrates, which have been developed for monitoring protein degradation by the UPS. These reporters are typically based on fluorescent proteins fused to degradation signals that target the fusion protein for constitutive turnover via the UPS (Neefjes and Dantuma, 2004). They include the ubiquitin-dependent reporters Ub^G76V^-green fluorescent protein (GFP) and GFPu that are targeted via an N-terminal ubiquitin or a short CL1 peptide motif, respectively (Dantuma et al., 2000; Bence et al., 2001). Another commonly used reporter, based on the degron derived from mouse ornithine decarboxylase (ODC), is degraded in a ubiquitin-independent fashion (Murakami et al., 1992). UPS reporter substrates are in principle devoid of intrinsic regulatory elements to eliminate the impact of variables such as posttranslational modifications, which can affect the half-life of endogenous protein substrates. Although all these reporter proteins are subject to degradation by the 26S proteasome, they are recognized by distinct targeting pathways and therefore differ in sensitivity and signal-to-background ratio. It has been shown that levels of the reporters inversely correlate with UPS activity (Dantuma et al., 2000; Bence et al., 2001). Accumulation of reporter protein relative to baseline levels is typically interpreted as reduced UPS function, however, reporter protein levels may be affected by changes in expression (Bowman et al., 2005; Tokui et al., 2009) or protein synthesis, to which they are very sensitive as a consequence of their short half-life (Li et al., 1998). These reporter substrates are important tools for studying UPS functionality in cell and mouse models of neurodegenerative diseases (Table 2).

**Table 2:** **Overview of measurements of the functionality of the ubiquitin/proteasome system in neurodegenerative disease models**.

**Disease**	**Protein context**	**Model system**	**Readout**	**Reduced function?**	**References**
Polyglutamine disease	Htt fragment	Yeast	Ub-R/P-lacZ; CPY*-HA	Yes	Duennwald and Lindquist, 2008
		HEK293 cell line	GFPu	Yes	Bence et al., 2001
			NES/NLS-GFPu	Yes	Bennett et al., 2005
			Ub^G76V^-GFP; Ub-R-GFP; GFP-CL1; ODC-GFP; GFP-dF508CFTR; TCRalpha-GFP	Yes	Hipp et al., 2012
		PC12 cell line	Ub-R/P-GFP; CD3delta-HA	Yes	Duennwald and Lindquist, 2008
			Ub^G76V^-YFP; YFP-CL1	Yes	Maynard et al., 2009
		R6/2 mice	GFPu	No	Bett et al., 2009
			Ub^G76V^-GFP	No	Maynard et al., 2009
		HD94 mice	Ub^G76V^-GFP	Yes^a^	Ortega et al., 2010
	Ataxin-1	HEK293T cell line	d2EGFP (ODC)	Yes	Park et al., 2005
		HEK293 cell line	NES/NLS-GFPu	Yes	Bennett et al., 2005
	Ataxin-7	Sca7^266Q/5Q^ mice	Ub^G76V^-GFP	No	Bowman et al., 2005
	Androgen receptor	HEK293 cell line	GFPu	Yes^b^	Mandrusiak et al., 2003
		NSC34 cell line	YFPu, NES/NLS-YFPu	Yes^c^	Rusmini et al., 2007
		AR121Q flies	GFPu	Yes^b^	Pandey et al., 2007
		AR97Q mice	Ub^G76V^-GFP	No	Tokui et al., 2009
Alzheimer's disease	β-amyloid peptide	B103 cell line	GFPu	Yes	Song et al., 2003; Oh et al., 2005
		HEK293 cell line	ZsProsensor (ODC)	Yes	Tseng et al., 2008
	Ubiquilin-1	HEK293T cell line	Ub^G76V^-YFP	No	Viswanathan et al., 2011
	APPswePS1dE9 mice		Ub^G76V^-GFP	No	Orre et al., 2013
			GFPu	Yes	Liu et al., 2014a
Parkinson's disease	α-synuclein	Yeast	GFPu	Yes	Outeiro and Lindquist, 2003
	LRRK2	HeLa cell line	Ub^G76V^-GFP	Yes	Lichtenberg et al., 2011
ALS	SOD1	NSC34 cell line	YFPu	Yes	Crippa et al., 2010; Onesto et al., 2011
			NES/NLS-YFPu	No	Sau et al., 2007
		C2C12 cell line	YFPu	No	Onesto et al., 2011
		SOD1G93A mice	Ub^G76V^-GFP	Yes^d^	Cheroni et al., 2009
ALS/IBMPFD	VCP	U2OS cell line	dF508CFTR	Yes	Weihl et al., 2006
		Meljuso cell line	Ub^G76V^-GFP; CD3delta-GFP	No	Tresse et al., 2010
Retinal degeneration	Rhodopsin mutant	HEK293 cell line	GFPu	Yes	Illing et al., 2002
			NES/NLS-GFPu	Yes	Bennett et al., 2005
		P23H, Rho^-/-^, and Rds mice	Ub^G76V^-GFP	Yes	Lobanova et al., 2013
	Transducin γ-subunit	G^-/-^_γ1_mice	Ub^G76V^-GFP	Yes	Lobanova et al., 2013
Prion disease	Prion protein	N2aPK-1 cell line	Ub^G76V^-GFP	Yes	Kristiansen et al., 2007
		Prion-infected mice	Ub^G76V^-GFP	Yes	Kristiansen et al., 2007

a *Transient UPS impairment*.

b *UPS impairment by mutant AR is ligand-dependent*.

c *UPS impairment by mutant AR occurs only in absence of ligand; ligand treatment restored UPS functionality*.

d *Reduced UPS activity observed only at late stages in cells which display advanced ALS pathology (e.g., enlarged vacuoles)*.

In cell culture experiments, an N-terminal fragment of polyQ-expanded huntingtin has been shown to impair the degradation of the endogenous substrate p53 (Jana et al., 2001) and GFPu (Bence et al., 2001). UPS inhibition appears to be a consequence of protein aggregation and not a unique feature of polyQ proteins since the unrelated aggregation-prone protein mutant cystic fibrosis membrane conductance regulator (CFTR) has a similar effect on the UPS (Bence et al., 2001). GFPu reporter accumulation correlates with induction of apoptosis, supporting a model in which UPS impairment induced by protein aggregation proteins is responsible for cell death although it cannot exclude that other adverse effects are responsible for the cytotoxicity (Bence et al., 2001). UPS impairment as a result of polyQ proteins affects both the nuclear and cytosolic UPS and does not seem to be confined to the compartment that accumulates the aggregation-prone proteins, arguing for general interference with UPS function (Bennett et al., 2005). A study utilizing a version of the GFPu reporter targeted to the synapse of neurons suggested that mutant huntingtin primarily affects UPS function in the synaptic compartment (Wang et al., 2008). It is noteworthy that later studies found the artificial CL1 degradation signal in GFPu to render proteins aggregation-prone (Menendez-Benito et al., 2005; Link et al., 2006), which is not surprising if one considers that this artificial degradation signal mimics targeting signals present in ER-derived proteasome substrates (Gilon et al., 2000). Subsequent studies have shown that the polyQ huntingtin fragment also affects the degradation of other UPS reporters, such as Ub^G76V^-GFP, signifying that this effect is not limited to aggregation-prone proteins (Maynard et al., 2009; Mitra et al., 2009; Hipp et al., 2012). Analysis in a cellular model demonstrates that aggregates of N-terminal huntingtin do not directly impair the proteasome, instead, the increase in ubiquitylated proteins likely reflects a general disturbance of the cellular proteostasis network (Hipp et al., 2012).

Two transgenic mouse models expressing GFP-based reporter substrates for the UPS, Ub^G76V^-GFP (Lindsten et al., 2003) and GFPu (Bove et al., 2006), have been instrumental in addressing UPS functionality in neurodegeneration *in vivo*. Using these mice, it has been demonstrated that UPS function is preserved in animals expressing polyQ-expanded proteins such as the N-terminal huntingtin fragment, R6/2 (Bett et al., 2009; Maynard et al., 2009; Ortega et al., 2010), androgen receptor (AR) responsible for SBMA (Tokui et al., 2009), and ataxin-7 that causes SCA-7 (Bowman et al., 2005). The lack of UPS impairment in the R6/2 mouse model has been particularly puzzling since the evidence for a globally dysfunctional UPS in cultured cells has largely been based on ectopic expression of the very same huntingtin fragment that is expressed in these mice (Bence et al., 2001; Jana et al., 2001; Bennett et al., 2007; Wang et al., 2008).

The development of quantitative mass spectrometry combined with efficient purification of ubiquitylated proteins or signature peptides from biological samples has been invaluable in undertaking detailed analysis of the ubiquitylated proteome in human diseases (Kessler, 2013). This method not only allows the determination of total ubiquitin levels, but also enables discrimination of different types of ubiquitin chains. Quantitative mass spectrometry has been successfully applied to investigate the composition of ubiquitin conjugates in R6/2 mice and HD patient brain and revealed accumulation of Lys11-, Lys48-, and Lys63-linked ubiquitin chains (Bennett et al., 2007). In theory, the increase in Lys11- and Lys48-linked ubiquitin chains would be consistent with a functional blockade of the UPS as both chain topologies can target proteins to the proteasome (Komander and Rape, 2012). However, Lys63-linked polyubiquitin chains also accumulate in HD and are not normally associated with proteasomal degradation (Nathan et al., 2013), suggesting that the effect of polyQ-expanded huntingtin on ubiquitin homeostasis is more complex than simple blockade of the UPS. In support of this view, two independent studies have not detected stabilization of Ub^G76V^-GFP (Maynard et al., 2009) or GFPu (Bett et al., 2006) in R6/2 mice. Ubiquitin conjugates observed in R6/2 brains have also been shown to differ qualitatively from those observed upon proteasome inhibition (Maynard et al., 2009), indicating that the observed increase in ubiquitylation in HD may be due to changes in ubiquitin-dependent processes other than proteasomal degradation.

While data obtained with mouse models for polyQ diseases have unequivocally supported the presence of a preserved UPS, UPS impairment has been detected in models of other neurodegenerative diseases. For example studies with the Ub^G76V^-GFP reporter mice have shown that UPS impairment occurs during progression in an ALS mouse model expressing mutant SOD1 (Cheroni et al., 2009). Likewise, UPS impairment has been demonstrated in a mouse model of prion pathology, which is consistent with a role for direct UPS impairment by the prion protein (Kristiansen et al., 2007). Opposing findings have been reported in the well-characterized APPswe/PS1dE9 mouse model for AD. A recent study has demonstrated accumulation of GFPu reporter as well as the endogenous proteasome substrate p53 in this model (Liu et al., 2014a), whereas the same mouse model did not accumulate Ub^G76V^-GFP according to another report (Orre et al., 2013). Although documented for some disorders, global inhibition of ubiquitin-dependent proteolysis does not appear to be a universal feature of neurodegenerative diseases.

## Compensatory mechanisms

The absence of functional impairment of the UPS in mouse models of polyQ diseases does not imply that the UPS is unaffected by the presence of aggregation-prone proteins. In cellular models, polyQ proteins have been shown to elicit UPS inhibition, indicating that they can have a general negative impact on intracellular proteolysis. Transgene expression levels are an important difference between the cell lines and mouse models. In cells, proteins are typically transiently overexpressed in an acute manner whereas proteins are expressed chronically at modest levels in transgenic mice, more similar to the situation in human patients. The effect of acute induction of the N-terminal mutant huntingtin fragment has been addressed *in vivo* (Ortega et al., 2010) using a mouse model in which huntingtin expression can be regulated by administration of doxycyclin (Yamamoto et al., 2000). Interestingly, acute overexpression of the mutant huntingtin fragment in these mice is accompanied by increased levels of the Ub^G76V^-GFP reporter substrate in neuronal and non-neuronal cells. Importantly, accumulation of the UPS reporter is of a transient nature, suggesting that cells activate adaptive responses to restore UPS activity (Ortega et al., 2010). Restoration of UPS function coincides with IB formation in neurons (Mitra et al., 2009; Ortega et al., 2010), which is consistent with a protective role of inclusions through sequestration of toxic oligomeric species. In support of this model, the Ub^G76V^-GFP reporter has been shown to accumulate also in R6/2 mice following treatment with chemical inhibitors of protein aggregation, which block the formation of IBs (Heiser et al., 2002; Ortega et al., 2010).

The autophagy pathway is tightly connected with the UPS and can play a compensatory role in maintaining intracellular protein degradation under conditions of reduced UPS activity. Autophagy counteracts the toxicity of mutant huntingtin, possibly through promoting the removal of aggregated, oligomeric species (Ravikumar et al., 2004). Moreover, autophagy induction reversed UPS impairment in a *Drosophila* model of SBMA (Pandey et al., 2007). Chemicals that inhibit lysosomal degradation also compromise the UPS, suggesting that autophagy is required for proper functioning of ubiquitin-dependent proteasomal degradation (Korolchuk et al., 2009). Autophagy likely assists the UPS in the removal of a pool of problematic proteasome substrates, such as aggregated or damaged proteins, which would otherwise impede UPS activity. It has been suggested that, analogous to UPS dysfunction in neurodegeneration, autophagy may be compromised in polyQ diseases (Ravikumar et al., 2004). Interestingly, experiments in mice have shown that autophagy dysfunction results in a neurodegeneration phenotype associated with ubiquitin-positive inclusions, indicating that an impaired autophagolysosomal system can recapitulate neurodegeneration (Hara et al., 2006; Komatsu et al., 2006).

**Figure 2:**
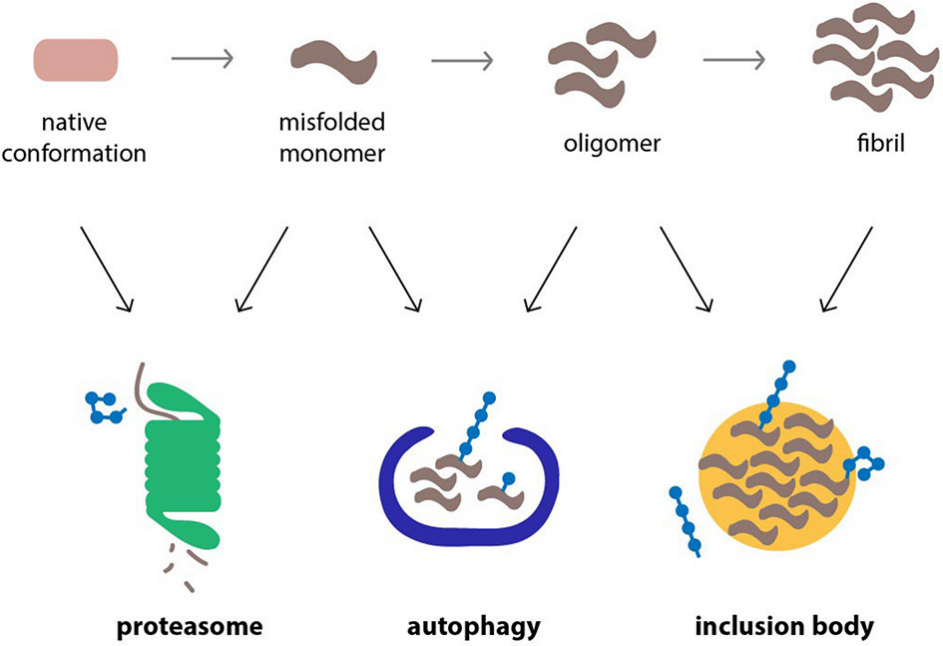
**Cellular pathways that counteract protein aggregation are ubiquitin-dependent processes**. Proteins linked to neurodegenerative diseases, such as α-synuclein, β-amyloid peptide and polyQ proteins, are prone to misfolding and aggregation in the cellular environment. The proteasome, autophagy, and inclusion bodies form a network of quality control systems which reduces levels of misfolded proteins and counteracts aggregation. All three pathways are regulated by ubiquitylation.

Ubiquitin, which serves a wide range of cellular functions besides proteasomal degradation, is closely involved in these compensatory mechanisms (Figure 2). It has been shown to target proteins to aggresomes by a mechanism that involves HDAC6, a cytosolic deacteylation enzyme that binds unanchored ubiquitin chains and facilitates the sequestration of aggregated proteins in IBs (Kawaguchi et al., 2003). The ability of specific adaptor proteins, such as p62 and NBR1, to simultaneously bind polyubiquitylated cargo and the autophagosome marker LC3 also supports a central role for ubiquitin in selective autophagy (Kraft et al., 2010). However, the role of ubiquitin as a decisive signal in autophagy has been questioned and instead it has been argued that protein oligomerization and not ubiquitylation is the primary signal (Riley et al., 2010). Both the formation of aggresomes (Olzmann et al., 2007) and ubiquitin-selective autophagy (Tan et al., 2008) are typically associated with Lys63-linked ubiquitin chains, although the involvement of other chain topologies is less clear. Notably, monoubiquitin can suffice as a signal to direct proteins to the autophagosome (Kim et al., 2008). More experiments are needed to dissect the functions of ubiquitin and the relative contribution of individual chain topologies in cellular processes.

The fact that ubiquitin is shared between targeting mechanisms that direct substrates to the proteolytic machinery and adaptive responses, which counteract toxic proteins, suggests a functional significance in directing the joint efforts of these pathways in eliminating harmful proteins (Groothuis et al., 2006). It is possible that the accumulation of polyubiquitin conjugates in polyQ diseases may be due to upregulation of ubiquitin-dependent adaptive responses, such as the formation of inclusions or activation of ubiquitin-selective autophagy, which through their actions may preserve UPS activity. Thus, rather than being a consequence of a functional impaired UPS, the accumulation of ubiquitin conjugates may be part of the reason why the UPS remains operative even in the challenging intracellular environment of polyQ disease.

## Reducing levels of neurodegeneration-associated proteins

In monogenic, dominant neurodegenerative disorders in which the identity of the disease-causing mutant proteins is known, as in the case of the polyQ disorders, an obvious but technically less trivial therapeutic approach would be to reduce the levels of the mutant protein. However, some of the gene products fulfill important functions in cells and may even be essential for viability, as is the case for huntingtin (Dragatsis et al., 2000) and ataxin-7 (Helmlinger et al., 2004). This problem may be circumvented by designing strategies to selectively target the mutated protein without affecting the wild-type form. Additionally, experiments in inducible mouse models for HD (Yamamoto et al., 2000) and SCA-1 (Zu et al., 2004) have shown that switching off expression of polyQ proteins reverses disease symptoms and neuronal pathology. A recent study has demonstrated that decreasing expression of polyQ-expanded ataxin-7 by 50 percent results in a full phenotypic rescue in a conditional SCA-7 mouse model (Furrer et al., 2013), which suggests that modest reduction of polyQ proteins may suffice for therapeutic intervention in humans. Targeting expression of mutant transcripts using RNA interference or antisense oligonucleotide technology is a promising approach to achieve this goal (Bonini and La Spada, 2005).

An alternative strategy for reducing levels of disease proteins is to accelerate their turnover by the UPS, whose function is preserved even at late stages of pathology. However, the question remains whether or not the proteasome can efficiently degrade disease-linked proteins. Some studies have suggested that the proteasome cannot degrade the polyQ proteins (Dyer and McMurray, 2001; Jana et al., 2001; Holmberg et al., 2004; Venkatraman et al., 2004) whereas others other reports show that they can be efficiently degraded by the proteasome as long as they remain in a soluble state (Verhoef et al., 2002; Kaytor et al., 2004; Michalik and Van Broeckhoven, 2004; Juenemann et al., 2013; Tsvetkov et al., 2013). Also, α-synuclein, expression levels of which can predispose individuals to PD (Singleton et al., 2003), can be degraded by the proteasome (Bennett et al., 1999), suggesting that other aggregation-prone proteins can be targeted by the UPS.

Several studies show that stimulating ubiquitylation and degradation of disease proteins through the UPS can rescue pathology in cell and animal models. For example, increasing the pool of free ubiquitin through genetic overexpression suppresses α-synuclein-induced neurodegeneration in *Drosophila* (Lee et al., 2009). The phenotypic rescue depends on the ability of the transgenic ubiquitin to form Lys48-linked polyubiquitin chains that typically target proteins for proteasomal degradation. Overexpression of certain ubiquitin ligases, which determine substrate specificity in the UPS pathway, also can confer protection in neurodegeneration models. Increasing levels of the ubiquitin ligase CHIP (C terminus of Hsc-70 interacting protein) delays the disease phenotype in SCA-1 (Al-Ramahi et al., 2006) and SBMA animal models (Adachi et al., 2007) through enhanced ubiquitylation and subsequent clearance of polyQ-expanded proteins. Moreover, Parkin has been shown to ubiquitylate ataxin-3 and reduces polyQ toxicity in cells (Tsai et al., 2003; Morishima et al., 2008). These findings show that the UPS is a plastic and versatile system that can be harnessed to accelerate the clearance of disease-linked proteins.

## Leveraging proteasomal degradation

**Figure 3:**
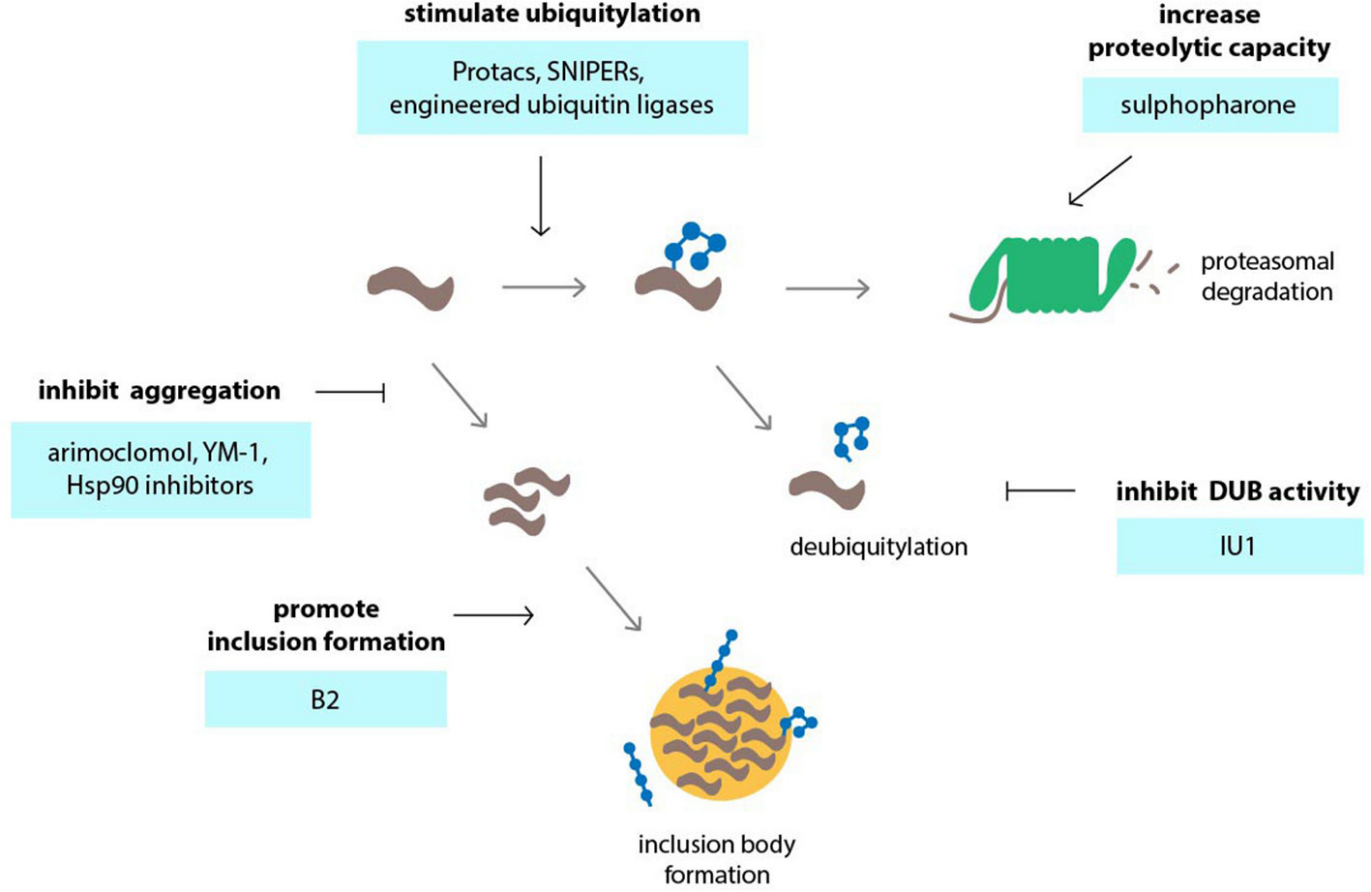
**Targeting the ubiquitin-proteasome system (UPS) in neurodegenerative disorders using small molecules or engineering approaches**. Various events in the UPS can be targeted by compounds in order to stimulate UPS activity. Among those events are accelerating of ubiquitylation by compounds or engineered ubiquitin ligases, inhibition of deubiquitylation, inhibition of protein aggregation so that the proteins remain in a state that is permissible to proteasomal degradation and stimulation of the formation of inclusion bodies which may reduce the load of aggregation-prone proteins and preserve UPS activity.

Augmenting UPS activity or targeting its activity toward disease-associated proteins may be achieved through small molecules and, though challenging, opens up the possibility of counteracting protein accumulation in neurodegeneration (Figure 3). One such strategy takes advantage of the fact that aggregation-prone proteins are better substrates for the UPS in their monomeric, soluble state compared to oligomeric assemblies. Compounds, which stimulate expression or activity of heat shock proteins, effectively counteract aggregation and increase the clearance of misfolded proteins through the UPS. The compound arimoclomol induces chaperone expression and has been shown to ameliorate disease in SBMA (Malik et al., 2013) and ALS mice (Kalmar et al., 2012). YM-1, a small molecule that increases the ability of the molecular chaperone Hsp70 to bind unfolded substrates, increases degradation of polyQ-expanded AR in cell culture and rescues toxicity in SBMA flies (Wang et al., 2013).

Stimulation of UPS activity is another means by which degradation of disease protein can be achieved. An important question is whether such a complex system involving a large number of proteins can be effectively activated by small molecules in a therapeutic setting. Recent studies have revealed that the capacity of the UPS can be regulated via two related transcription factors, Nrf1 and Nrf2. Nrf1 is synthesized as an ER-anchored protein and only becomes transcriptionally active in the nucleus when increased UPS function is required (Radhakrishnan et al., 2010; Grimberg et al., 2011). Nrf2 regulates the antioxidant response in cells and can stimulate expression of proteasome subunits, which likely increases degradation of oxidized proteins during stress (Pickering et al., 2012). Nrf2 can be induced by the small molecule sulforaphane, which increases proteasome levels and activity (Kwak et al., 2003, 2007), and enhances UPS function *in vivo* (Liu et al., 2014b). Sulforaphane has been shown to reduce mutant huntingtin protein and mitigate polyQ toxicity in neuronal cells (Liu et al., 2014b).

Ubiquitin-dependent proteasomal degradation of aggregation-prone proteins can also be stimulated through direct modulation of proteasome function (Lee et al., 2010). The regulatory particle of the proteasome harbors two deubiquitylation enzymes, namely UCH-L5 and USP14/Ubp6, that counteract proteasomal degradation by trimming ubiquitin chains of recruited substrates (Finley, 2009). It is has been proposed that chain trimming by proteasome-associated deubiquitylation enzymes may function as a molecular timer that determines the time window during which the proteasome can initiate degradation, thereby rescuing poorly ubiquitylated proteins from degradation (Lam et al., 1997). IU1, a selective small molecule inhibitor of USP14, has been shown to accelerate proteasomal degradation of aggregation-prone proteins, including proteins associated with neurodegenerative diseases such as tau, TDP43 and the polyQ protein ataxin-3, as well as oxidized proteins (Lee et al., 2010). However, it remains to be seen whether IU1 is effective in mitigating disease manifestations in animal models of neurodegenerative disorders.

## Selective proteasomal targeting of disease proteins

Increasing overall degradation by stimulating UPS activity may be accompanied by unwanted side effects due to their general nature. Selectively targeting the disease-causing proteins for proteasomal is therefore expected to have many advantages over general stimulation of UPS activity. The specificity of the UPS action towards disease-linked proteins can be increased by taking advantage of the natural regulatory systems that dictate the degradation rates of these proteins. While this method has the potential of reducing side-effects of increasing overall UPS activity, it requires detailed insights in the biological function and regulation of the disease-causing protein.

Among the proteins implicated in polyQ diseases, AR is probably the one whose functions are best understood. AR is a nuclear hormone receptor that is linked to various types of cancers, most notoriously prostate cancer (Matsumoto et al., 2013). The idea of using small molecule inhibitors of heat shock protein 90 (Hsp90) as therapeutic tools in SBMA originates from cancer studies which revealed that AR is an Hsp90 client and requires heat shock proteins for proper functioning (Prescott and Coetzee, 2006). It has been shown that geldanamycin analogs 17-AAG and 17-DMAG accelerate degradation of AR and other Hsp90 client proteins and reduce polyQ-expanded AR toxicity in cell and mouse models (Waza et al., 2005; Tokui et al., 2009). Interestingly, geldanamycin is also effective in preventing aggregation of mutant huntingtin, which has been attributed to the ability of Hsp90 inhibitors to induce a general heat shock response (Sittler et al., 2001). Activation of the heat shock response may also contribute to lowering of AR half-life by geldanamycin analogs in SBMA models, as overexpression of heat shock proteins has been shown to also promote AR degradation (Bailey et al., 2002). Small molecules that promote the deposition of aggregation-prone proteins into IBs may also be beneficial in neurodegenerative diseases. For example, the compound B2 has been shown to increase inclusion formation of the mutant huntingtin fragment, polyQ-expanded AR, and α-synuclein, and thereby suppresses toxicity in cell and fly models (Bodner et al., 2006; Palazzolo et al., 2010).

Protein stability can be influenced by posttranslational modifications. One example for this is again AR, whose levels and subcellular localization is controlled by the PI3K-Akt signaling pathway. It has been shown that phosphorylation of mutant AR by the Akt kinase, which can be induced by insulin-like growth factor-1 (IGF-1), accelerates clearance of the receptor by the proteasome (Palazzolo et al., 2007). Importantly, IGF-1 reduces mutant AR aggregation and toxicity in SBMA models *in vitro* and *in vivo* (Palazzolo et al., 2009; Rinaldi et al., 2012). More recently, it has been shown that the turnover of ataxin-1 is regulated by phosphorylation by MSK1 and, accordingly, the levels of polyQ-expanded ataxin-1 could be reduced by depletion of MSK1 (Park et al., 2013). The phosphorylation event is controlled by components in the MAPK-Ras pathway, and pharmacological curtailing of the MAPK pathway with small compound inhibitors of MEK1/2 or Raf1 similarly reduced levels and toxicity of mutant ataxin-1 (Park et al., 2013).

In recent years, scientists have begun to explore the possibility to mobilize branches of the UPS that are not normally involved in handling aggregation-prone proteins. Earlier studies have shown that engineered chimeric ubiquitin ligases can be used to redirect the ubiquitylation machinery to proteins of interest (Zhou et al., 2000). Engineered ubiquitin ligases typically contain two domains: a recognition domain specific to the protein of interest, and a ubiquitylation domain. Examples for the latter are RING, HECT, and U-box domains, which directly catalyze the addition of ubiquitin to the substrate. Ubiquitylation domains may also guide the substrate protein to the enzymatic activity of a ubiquitin ligase, such as the SCF complex via the F box domain (Zhang and Zhou, 2005). An example of engineered ubiquitin ligases in the context of neurodegenerative diseases is a chimeric Dorfin-CHIP fusion that combines the substrate binding domain of the ubiquitin ligase Dorfin with the U-box of CHIP. The resulting fusion protein was found to ubiquitylate mutant SOD1 more efficiently than the wild-type Dorfin or CHIP ubiquitin ligases (Ishigaki et al., 2007). Off-target effects due to residual activity toward their native substrates, or unwanted regulation in the cellular microenvironment are a major caveat with current chimeras based on domains derived from endogenous proteins. However, an important advantage of the chimeras is that they can be designed with high specificity to certain sub-populations of the protein-of-interest. For instance, a recent study reported the generation of an SCF-βTrCP ubiquitin ligase that selectively targets only activated ErbB receptor tyrosine kinases for degradation (Kong et al., 2014).

Nanobody-based fusions are variations on protein chimeras based on single chain antibodies, which, when expressed in the intracellular environment, bind to target proteins with high specificity (Muyldermans, 2013). A fusion consisting of an F-box domain and a nanobody directed to GFP has been successfully used to deplete cytoplasmic, nuclear, and transmembrane GFP-fusion proteins in cultured cells and *in vivo* via the SCF complex (Caussinus et al., 2012). In theory, this approach may also be used to engineer intrabody-based ubiquitin ligases specific to disease-associated proteins, or even to pathogenic protein conformations. Although engineered ubiquitin ligases can be of great value in experimental settings and provide us with a proof-of-principle that ubiquitylation can be tailored to accelerated degradation of desired proteins, there are currently many practical limitations that limit their adaptation to therapeutic settings, such as safety and delivery to target tissues.

A number of studies have explored whether small molecules can be used to redirect endogenous ubiquitylation enzymes to defined target substrates. Most interesting in this respect is the work on molecules known as Protacs (Proteolysis-targeting chimeric molecules) and SNIPERs (Specific and non-genetic IAP-dependent Protein Erasers) (Buckley and Crews, 2014). Both types of molecules combine a ubiquitin ligase interaction peptide with a small molecule ligand specific for the target protein (Sakamoto et al., 2001). Protacs have mainly been developed to target cancer-relevant gene products but may be applicable as well to misfolded proteins in neurological disorders. One example is a chimeric molecule that unites a peptide motif from HIF1α with the AR ligand dihydrotestosterone (DHT) and can be used to accelerate degradation of AR in cultured cells (Rodriguez-Gonzalez et al., 2008). Although it would be interesting to test whether this molecule can also accelerate the degradation of mutant AR in SBMA or whether derivatives can be generated for other polyQ proteins, a limiting factor in the development of Protacs is poor permeability across cell membranes and potential antigenicity due to their peptidic nature. However, in the cases where the ubiquitin ligase can be recruited by small synthetic molecules instead of polypeptides, it may be possible to obtain compounds with potential therapeutic value. A functional cell-permeable Protac combines two active molecules, the AR ligand DHT and the Mdm2-interacting compound Nutlin, to increase ubiquitylation of AR by Mdm2 (Schneekloth et al., 2008). In SNIPERs, an IAP1-interacting bestatin ester replaces the peptide domain found in Protacs that links the chimeric molecule to ubiquitin ligase activity. SNIPERs have been used successfully in the context of steroid receptors, including AR, and efficiently reduce receptor protein levels at lower molar concentrations than Protacs (Okuhira et al., 2013). Small molecules for targeted degradation of disease-associated proteins by the UPS have so far mainly been explored in the cancer field, but the successful application in malignant cells may serve as a proof-of-principle to warrant their investigation in neurodegenerative disorders.

## Concluding remarks

Increasing evidence suggests that ubiquitin-dependent proteolysis is largely operative in many neurodegenerative diseases, and hence a causal relationship does not exist between aggregation-prone proteins and global UPS impairment. While perturbations in UPS function due to disease proteins cannot be excluded, adaptive responses such as IB formation and autophagy likely contribute to the restoration of cellular protein homeostasis. This knowledge justifies further exploration of the protein degradation machinery for treating or preventing debilitating neurodegenerative disorders. However, in order to fully appreciate the potential of the UPS as a therapeutic target, we need to continue to decipher how the UPS and compensatory pathways coordinate the detoxification and clearance of misfolded proteins.

While the current data do not support a scenario in which global impairment of the protein quality control function of the UPS lies at the basis of proteinopathies, our present understanding strongly supports an involvement of ubiquitin-dependent processes in the development or progression of neurodegenerative diseases. Dissecting the molecular mechanisms and key players responsible for changes in ubiquitylation may help to identify suitable therapeutic targets. Because it appears to be unlikely that this inhibitory activity is a shared intrinsic feature of aggregation-prone proteins, it will be important to study the interplay between the UPS and the individual disease-associated proteins in the context of their native functions.

This change of perspective from the UPS as a potential cause for neurodegeneration to it being a preserved proteolytic pathway that may be exploited in therapeutic approaches has been an important contribution of the large number of studies that have probed into the role of the UPS in polyQ disorders. Inspiring examples can be found in the identification of compounds which stimulate UPS activity and the development of small molecules that target desired proteins for proteasomal degradation. Although these studies provide us with proofs-of-principle for redirecting proteins to endogenous ubiquitin ligases, there is a clear need for compounds with more favorable drug-like properties in order to accomplish this aim in a physiological setting. If successful, such small molecules could be used on their own or in combination with other polyQ protein-reducing therapies, such as RNA interference, to reduce the levels of the toxic proteins in patients. Despite the fact that much needs to be done before we can start to evaluate the applicability of such approaches, the recent insight that at least the UPS is still on our side in fighting the toxic effects of the these rogue proteins opens other opportunities in the pursuit for therapeutics for neurodegenerative diseases.

### Conflict of interest statement

The authors declare that the research was conducted in the absence of any commercial or financial relationships that could be construed as a potential conflict of interest.
